# Assessment of modern contraceptives continuation, switching and discontinuation among clients in Pakistan: study protocol of 24-months post family planning voucher intervention follow up

**DOI:** 10.1186/s12913-018-3156-0

**Published:** 2018-05-11

**Authors:** Moazzam Ali, Syed Khurram Azmat, Hasan Bin Hamza

**Affiliations:** 1 Department of Reproductive Health and Research, World Health Organization, Geneva, Switzerland; 2 Department of Uro-Gynaecology, University of Ghent, 9000 Ghent, East Flanders Belgium; 3 Department of Paediatric Emergency Medicine, The Hospital for Sick Children, Toronto, Ontario Canada; 4 Public Health Consultant, Islamabad, Pakistan

**Keywords:** Vouchers, Family planning, Contraception, Continuation, Switching

## Abstract

**Background:**

Pakistan has the second highest fertility rate in South Asia and its increasing population growth presents a significant challenge for country’s path to progress and development. Modern contraceptive methods only account for a slow-rising 26% of use in Pakistan which is further lowest in the underserved areas (< 20%), with a high unmet need for family planning (20%). The David and Lucile Packard Foundation USA and Pakistan funded two operational research projects from 2012 to 2015, that employed a Demand-side Financing (DSF) approach testing the effectiveness of single and multi-purpose voucher schemes in increasing access and uptake of FP services and products among the women of two-lowest income quintiles in the Punjab province of Pakistan. The present paper presents a study protocol which intends to assess the longer term impact of these two voucher intervention programs among married women of reproductive age (MWRA) who received contraceptive services through vouchers.

**Methods:**

This will be a mixed methods study using qualitative and quantitative approaches. A quantitative cross sectional survey will measure the contraceptive uptake among voucher users, included in the endline survey and to examine the attitudes and behaviour of women with respect to contraceptive continuation, switching and discontinuation 24 months post intervention in two districts of Chakwal and Faisalabad in Punjab province of Pakistan. Qualitative in-depth interviews will be conducted with FP service providers operating in intervention areas and with key policy makers in the public sector to examine and document the service provider perspective on sustainability on contraceptive practices and behaviour in the post project closure period within the intervention areas.

**Discussion:**

Globally, there is almost negligible direct evidence on the assessment of longer-term impact of a demand-side financing programs using free or subsidized vouchers for family planning services especially during post-intervention period or when donor money runs out. The findings of this study will help fill the knowledge gap in the context of sustainability issues post-intervention and will provide information to policy makers to develop and plan contraceptive services in the target area to sustain the positive behaviour change in the population.

**Electronic supplementary material:**

The online version of this article (10.1186/s12913-018-3156-0) contains supplementary material, which is available to authorized users.

## Background

Pakistan has the second highest fertility rate (3.8) in South Asia after Afghanistan (5.3) [1, 2] and its increasing population growth presents a significant challenge for country’s path to progress and development. Family planning has been a contentious issue in Pakistan for decades. In spite of almost universal prevalence of knowledge about family planning methods, the overall contraceptive use is very low – 35%, where one of the major contributors among the method-mix is the use of traditional methods [1, 2]. Modern contraceptive methods, which have been documented to be highly effective means of preventing pregnancies in order to ensure healthy timing and spacing of births [3], only account for a slow-rising 26% of use in Pakistan which is further lowest in the underserved areas (< 20%), with a high unmet need for family planning (20%) [2].

On average, Pakistani women have four children during their lifetime – one more than they would like. In addition, Pakistan also shows very little uptake of long acting reversible contraceptive methods while short term and permanent methods have greater utilization [2, 4]. For example, female sterilization/tubal ligation is the most common method at around 45% of the modern method mix, but it is chosen late, often after 31.5 years of age and usually after four or more children. Second, short-term methods such as condoms accounted for around 23% of the method mix, with the remainder divided between the pill, injection, and long-term reversible method (LARC) for contraception e.g., Intrauterine Contraceptive Device (IUD). In addition, a wide gap between the knowledge (99%) and practice of modern contraceptives was documented in the last demographic survey.

A national program for family planning was launched under the auspices of the Government of Pakistan in the late 1950s but it has suffered from insufficient financing, de-motivated human resources, inefficient information system, non-responsive service delivery, and weak contraceptive supply chain system [6]. The government is still considered the main FP service provider (coverage to 45% of the population), but its expenditures on health and population continue to be very low. Even though, FP services are offered at public sector facilities/government centres for free; however, the utilization is low due governance challenges, including provider absenteeism, dual practice, less motivation; frequent stock-outs, maintenance of the equipment and facility; and sometimes exorbitant informal fees, [7, 8] therefore people resort to private sector where they find better quality and responsive services, although with a significantly higher price tag attached [9].

The private sector plays an important role in health care services. Pakistan’s non-state providers or private-sector providers deliver 35% of current services to FP users, and a further 15.5% are delivered by other private health practitioners (e.g., midlevel female health visitors, traditional birth attendants, pharmacists, traditional healers, shops) [2].

Considerable global and local experience indicates that the private sector can make services available, provide quality of care to users and, where programs are appropriately designed, reach poor and vulnerable women and girls [10]. Despite the fact that private sector is having larger share in FP service provision, yet it is not geared to provide FP as a front line service because of low margin of profit on contraceptive commodities and consultation as compared to other clinical and diagnostic services, where it makes far more profit [11, 12]. CPR is stubbornly low low despite considerable demand and knowledge of FP methods, unmet need remains high, particularly among the poor. Social pressures, particularly in poorer communities, call for women to have numerous children. Thus, permanent contraception (predominantly tubal ligation) is chosen only after an average 4–5 children, and use of long-acting methods (LARC) is negligible (~ 2%). Inequities are also noted in access and use of family planning services, where access to funds remains one of the key reasons for poor women for not using FP services bedsides other factors in Pakistan [2, 13–15].

Given the scale of unmet need for family planning services in Pakistan, there is adequate space to support the scale-up of both state and non-state provision, and the taking of a “total market” approach to RH and FP service provision to avoid crowding out existing provision [16]. The social marketing and social franchising aspect is also unlikely to crowd out public sector provision but should increase overall demand, considering the scale of current unmet need.

As part of efforts in facilitating the government of Pakistan in achieving national development goals of improving maternal and reproductive health by increasing modern contraceptive method uptake, the David and Lucile Packard Foundation funded two operational research projects [17, 18] from 2012 to 2015, that employed a Demand-side Financing (DSF) approach testing the effectiveness of single and multi-purpose voucher schemes in increasing access and uptake of FP services and products among the women of two-lowest income quintiles in the Punjab province of Pakistan [19, 20]. The first project was implemented by Marie Stopes Society (MSS) Pakistan, and tested how effective a free single-purpose voucher scheme would be in enhancing FP access and use [19, 21, 22]. The second project was implemented by Green Star social marketing Pakistan (GS)/Population Services International (PSI) and tested the effectiveness of a subsidized multi-purpose voucher scheme on increase access and use of FP and other Maternal Child Health (MCH) services use (20)^2^. The research employed quasi experimental methods employing baseline and end-line household surveys (Refer to Additional file 1) before and after the implementation of interventions [23–25] (Table 1).

**Table 1:** Reproductive Health Indicators: comparison of 2006–07 & 2012–13 demographic health surveys [2, 5]

Key Indicators	PDHS 2006/07	PDHS 2012/13
Total fertility rate (per women)	4.6	3.9
Maternal mortality ratio (per 100,000 live births)	276	178
Teen pregnancy (%)	9	8
CPR (any method) among currently married women (%)	30	35
Unmet need for family planning (%)	25	20

### Overview of single versus multi-purpose voucher intervention projects in Pakistan

Although the single (MSS study) and multi-purpose voucher (GS/PSI study) interventions were effective in increasing contraceptive use, however the lack of willingness among women was noted in continuing to use contraceptives in the absence of vouchers presents programmatic challenges for future scaling up of voucher schemes (Refer to Additional file 1) [24]. It has been noted previously that an increase in spacing through contraceptive use is beneficial for improving maternal health [26–28]. The findings from the two studies i.e. MSS and GS/PSI from Pakistan reported the IUD discontinuation rate of 18.8 and 19.4% after insertion, respectively at 12 months and the women who discontinued IUD use, almost 57% of them did not switch to another method which is significantly lower than the national trend of 26% [21, 29, 30]. Nearly half of them cited desire to become pregnant as a reason for not switching to another method. Globally, there is almost negligible direct evidence on assessment of longer-term impacts of a demand-side financing programs using free or subsidized vouchers especially during post-intervention period or when money runs out. Although, a recent evaluation capturing the longer-term impacts of a 2 years unconditional cash transfer (UCT) program from Malawi documented that the intervention group (young women) who were exposed to UCT, the short-term benefits of the program were mostly dissipated [31]. For example, there was a delay in age of marriage, low fertility and low incidence of HIV infection observed during the intervention; however, the immediate post-intervention period noted sharp rise in marriages followed by pregnancies.

The proposed research project intends to assess the longer term impact of voucher intervention programs among married women of reproductive age who received contraceptive services through vouchers. The research will study the pattern of current modern contraceptive method use among voucher users 24 months post-voucher intervention. These projects ended in March 2015. We, therefore, propose to conduct a cross sectional survey to ascertain patterns of current modern contraceptive continuation, switching and discontinuation, and fertility behaviours among voucher users twenty-four months after the end of intervention. We would also like to conduct qualitative in-depth interviews (IDIs) with project services providers in order to document their perspectives on appealing and discouraging aspects on the service provision model; quality of care; motivations; experience, challenges and opportunities concerning the provision of contraceptive services. In addition, few IDIs will also be conducted with key public sector stakeholders to capture their view point on such project outcomes, their endline evaluation recommendation and utility for policy and practice.

The purpose of this study is to collect information on FP practices of voucher users outside the parameters of a ‘project framework’ that ended twenty-four months back. It is essential to ascertain that in the absence of voucher facilities and programmatic impetus what happens to user behaviours, if they continue, discontinue or switch the use of FP products or services. We also wanted to understand the enablers and bottlenecks in FP continuation outside the parameters of the FP intervention project. The findings of this study will help fill the knowledge gap in the context of sustainability issues after the intervention finishes and will provide information to policy makers to develop and plan contraceptive services in the target area to support and sustain the positive behaviour in the population.

The study objectives are:

#### Users perspective

1. To measure the CPR among voucher users, included in the endline survey, twenty-four months post intervention, in districts of Chakwal and Faisalabad, Pakistan.
2. To examine the attitudes and behaviour of women with respect to contraceptive use, post intervention, in districts of Chakwal and Faisalabad, Pakistan.

#### Provider’s perspective

3. To examine and document the service provider perspective on sustainability on contraceptive practices and behaviour in the post project closure period within the intervention areas

#### Policy maker’s perspective

4. To ascertain any progress at the public sector policy making level with respect to endline survey project recommendations shared with key donors, implementing agencies and national policy makers

### Ethics approval

The Ethics approval was obtained from the WHO Research Ethics Review Committee under the project ID: A65911.

## Methods

### Study design

This will be a mixed methods study. A cross sectional survey will be conducted to meet objectives 1 and 2. The cross-sectional survey will recruit all voucher users included in the end line survey. At the time of the end line survey we developed a separate but detailed list of voucher users in both intervention areas and we intend to use the same database to recruit voucher users for the proposed survey. Objectives 3 and 4 will be addressed by using qualitative research methods. In depth interviews (IDIs) will be conducted with FP service providers operating in intervention areas and with key policy makers in the public sector to meet objectives 3 and 4.

### Sampling procedures for the quantitative survey

For the cross-sectional survey we plan to approach all 800 voucher users who were part of the end line survey in March 2015. The sampling frame of the voucher users is available for both MSS and GSM components and the basis of sample size distribution is as follows:

#### Sample size

Sample size has been calculated separately for MSS and GS components using the frequency of ‘current contraceptive use’, from the respective endline surveys, as the primary indicator. PASS.v.11® has been used for sample size calculation.

#### MSS component

A sample size of 257 from a group of 400 voucher clients will achieve 80% power to detect a 5% difference (P1-P0) using a two-sided binomial test. The target significance level is 0.05. The proportion under the null hypothesis is 0.51, based on the frequency of ‘current contraceptive use’ taken as 51% from the endline survey.

#### Greenstar component

A sample size of 268 from a group of 400 voucher clients will achieve 80% power to detect a 5% difference (P1-P0) using a two-sided binomial test. The target significance level is 0.05. The proportion under the null hypothesis is 0.43, based on the frequency of ‘current contraceptive use’ taken as 43% from the endline survey.

In addition, information will be collected via a face to face interview using a short questionnaire. Information on the following issues will be collected:

- past and current contraceptive use,
- switching
- reasons, if discontinued use,
- first-time FP users
- difficulties in continuing contraceptive use – supply issues
- pregnancies or births experienced to ascertain method failure or discontinuation
- role of cost in use of any modern method

### Sampling procedure for the qualitative survey

We plan to interview 10–15 providers from MSS project areas, the same number of providers will be interviewed in GS project areas. We also plan to include policy makers who have taken part in voucher programme implementation, and are from district or provincial administration. The number of policy makers to be interviewed will be around 10–12.

### Study site(s)

The study will be conducted in intervention districts MSS and GS-PSI project in Chakwal and Faisalabad of Punjab province, Pakistan.

### Study participants

We plan to interview voucher users recruited in the end line surveys conducted for the “Assessment of effectiveness of two demand side financing voucher schemes in meeting birth spacing needs of the underserved in Punjab, Pakistan” project [24, 25]. FP service providers operating in intervention areas of the above project will be eligible for participation in the study. Additionally, for policy maker interviews, federal and provincial government policy experts will be approached for IDIs.

### Inclusion criteria

1. Voucher users will be included in the study if they are married and between 15 and 49 years of age.
2. FP service providers will be included in the study if they were providing the services for the project at the time of the endline survey
3. Key policy makers will be included in the study if they are in a decision making position within the public sector and with whom endline survey project recommendations were shared

## Data management and analysis

### Data collection

Quantitative data will be collected via structured questionnaire. A list of voucher users who were interviewed for the endline survey is available and we plan to contact the same voucher users through their respective providers due to cultural sensitivities issues. The voucher users thus available will be requested to answer questions on current and past contraceptive, method discontinuation and switching, as well as attitudes and perceptions on contraceptive usage.

Qualitative data collection will be conducted based on information collected through IDIs with FP service providers and key policy makers. The interviews will be tape recorded, transcribed and translated into English where needed. Data will be analyzed using descriptive and qualitative content analysis. Transcripts of interviews and notes will be analyzed for significant themes that are identified keeping in mind the study objectives 3 and 4. Both ‘manifest content’ (visible, obvious components) and ‘latent content’ (underlying meaning) of the text will be analyzed.

### Data analysis

Quantitative data from the cross-sectional survey will be entered into an electronic database using Visual FoxPro 6.0 and Epidata 3.1. All data will be double entered into existing software used for the endline date by two separate data operators. Measures will be taken to ensure the quality of collected data. All the forms will be checked on a daily basis for completeness, logical errors, and unclear or irrelevant responses. Monitoring visits will also be made by the Principal Investigator, co-investigators and project team to ensure the quality of data and adherence to the study protocol. If needed, refresher training will be arranged to emphasize understanding of data collection tools. Primary data will be analysed using descriptive and inferential statistics collected from the voucher users through the survey. Descriptive analysis will encompass computing the frequencies and proportions of categorical variables while mean and other measures of central tendency will be computed to describe continuous variables. Main outcome variables will be - past and current contraceptive use (by method), switching and discontinuation with reasons. We will compute prevalence rates for the main outcome variables and also conduct analyses to determine their associated factors using univariate and multivariable analysis through logistic regression techniques.

For qualitative analyses transcripts will be read several times to understand in depth the respondents’ experiences, their views. ‘Meaning units’ that mirror statements, will be then identified as per topic guide by highlighting phrases in the transcripts which will be ‘condensed’ and thereafter ‘codes’ will be identified from the ‘condensed meaning units’ without losing the context. Finally, the research team will review the codes independently and will group similar codes into sub-categories and categories. From the categories theme and sub-themes with be identified after systematically analysing the commonalities, variations and disagreements. We will further analyse data with a focus on description and interpretation of message meaning and concepts to validate the findings of the study.

### Project timeline

The overall duration of the study will be as follows:

**Table:** 

Milestone	Calendar
Concept note	August 2016
Research proposal	September 2016
Tool development	September–October 2016
Ethics and scientific approval	November – February 2017
Identification of team and training	March–April 2017
Data collection	May –July 2017
Data entry	July – August 2017
Analysis	August–September 2017
Report writing	September – October 2017
Dissemination	December 2017

### Dissemination

The results will be published in peer-reviewed journals, and presented at national and international meetings and conferences. Briefs indicating policy and programmatic implications of findings will be produced, and sessions will be organized involving professional societies and government officials in discussions on translating evidence into action.

Results are expected to guide policy makers, managers and other stakeholders in the development of measures post-health interventions to sustain the positive behavior change in the population. The evidence generated by the study will contribute to better understanding of longer-term impact of a demand-side financing programs in contraception.

### Main problems anticipated and proposed solutions

We expect that participants of this study do not face any risks that may result in harm or injury. However there is there is some potential for loss of confidentiality that may occur during the study. To address this all study staff will be trained to protect the confidentiality of participants to the fullest extent possible. All personal information such as participants’ contact details and informed consent with participants’ names will be stored under lock and key. Personal identifiers will not be published and not be included in data analysis. Any data and reports sent to funding organization will have the personal information removed.

### Ethical considerations

Measures will be taken to ensure high ethical standards for the quality of collected data. All the forms will be checked on a daily basis for completeness, logical errors, and were cleared of irrelevant responses. Monitoring visits will be made by the principal investigators (PIs) and co-investigator to ensure the quality of data and adherence to the study protocol. Refresher training will be arranged to emphasize understanding of data-collection tools. Software will be developed for data entry restricting to mandatory fields and extreme values. Hard copies of completed tools (questionnaires, checklists, reporting formats) will be kept in locked storage before and after data entry. No financial incentives will be provided to the study participants. No personal information will be documented on the study tool which can link data to the respondents. Steps will also be taken to ensure confidentiality during interviews.

## Discussion

The current evidence suggests that demand-side financing vouchers for FP likely increases use of modern contraceptive methods [24–27, 32]. By targeting underserved groups, vouchers ensure subsidies reach the disadvantaged and are not absorbed by those with greater access to resources [33, 34]. In addition, vouchers can be considered as a stepping-stone toward social health insurance, for example vouchers may help governments develop their capacity to purchase health services (accreditation, pricing, contracting, quality assurance, monitoring, claims processing, and reimbursement) and to target subsidies to underserved populations. In the Philippines, the government and the World Bank are working to integrate vouchers with the Philippines Health national insurance scheme [35]. Unfortunately, in Pakistan the recently launched national health insurance scheme does not list FP as an offered service.

However, there is a lack of information on what happens to outcomes such as CPR or contraceptive method continuation once the implementation via a specific project ends especially when majority of implemented projects are either target or projection-based not impact oriented. This information will be essential for government/donors in reaching an understanding vis-à-vis the sustainability and financial security of FP programmes once such DSF-based voucher schemes have ended. This new information is considered to help in developing an understanding of the equity-based impact of voucher interventions after the withdrawal of funding from funding agencies, as mainly donors leave after three to 5 years. Such an understanding will have wider implications with respect to future steps that the government may need to take in order to improving maternal and child health. The information to be collected has the potential to provide empirical evidence for FP intervention models that are sustainable and are reaching to those in need of services.

## Additional file

Additional file 1: Summary of the findings from the two projects [24]. (DOCX 30 kb)

## Abbreviations

CPR: Contraceptive prevalence rate
DSF: Demand-side financing
FP: Family planning
GS: Green star, Pakistan
HIV: Human immunodeficiency virus
IDI: In-depth interviews
IUD: Intrauterine contraceptive device
LARC: Long-term reversible method
MCH: Maternal child health
MSS: Marie stopes society, Pakistan
MWRA: Married women of reproductive age
UCT: Unconditional cash transfer
WHO: World Health Organization
