# Supplementary material for: Assessment of modern contraceptives continuation, switching and discontinuation among clients in Pakistan: study protocol of 24-months post family planning voucher intervention follow up
Source: BMC Health Serv Res. 2018 May 11;18:359. doi: 10.1186/s12913-018-3156-0 (PMC5948711; doi:10.1186/s12913-018-3156-0)
Supplement: Supplementary file 1 — Summary of the findings from the two projects [24]. (DOCX 30 kb) [file 12913_2018_3156_MOESM1_ESM.docx]

**Additional file 1 Summary of the findings from the two projects (24)**

***Single purpose voucher impact***

The findings from the MSS study’s, evaluation suggest that provision of a free single purpose voucher is highly effective in increasing modern contraceptive use among women during the course of the project up to 31% in intervention (baseline: 19%, endline: 50%) and 6% (baseline: 16%, endline: 22%) in control areas. This translates into a net increase of 26% in modern contraceptive uptake. Though all modern contraceptives demonstrated an increase, however interestingly, use of Intra Uterine Devices (IUDs) showed promise which increased by a net 16%, especially among women from the poorer socio-economic segments. . Majority (91%, n=390) of the voucher users reported using a contraceptive method while the most common method being used by voucher users was IUD (53.6%) (Refer to Table S1). Approximately63%, (n=348) of the voucher users were first time contraceptive users. A majority of the voucher users (84%) expressed unwillingness with respect to future intentions to use FP services in the absence of a voucher scheme.

**Table S1: Contraceptive method mix for voucher users receiving family planning services through the MSS single-purpose voucher scheme – 24 months period**

| **Method** | **Frequency (number of voucher clients)** | **Percentage** |
| --- | --- | --- |
| Pill | 20 | 5.1 |
| IUD | 209 | 53.6 |
| Injections | 40 | 10.3 |
| Implants | 29 | 7.4 |
| Condom | 51 | 13.1 |
| Female sterilization | 3 | 0.8 |
| Lactational amenorrhea method | 2 | 0.5 |
| Non user | 36 | 9.2 |
| Total | 390 | 100.0 |

***Multipurpose purpose voucher impact***

Likewise, the use of subsidized multi-purpose vouchers in study implemented by GS/PSI was found to be effective in increasing modern contraceptive use by 15% among women in intervention group during the course of the project. Among modern methods condom use increased by a net 4%. Majority (87%, n=409) of the vouchers clients reported using a contraceptive method while the most common method of contraception being used by voucher users was IUD (30.8%) followed by Injections (22.5%) (Refer to Table S2). Almost two-third (67%, n=343) voucher users using a modern method also reported using the contraceptive for the first time. On future intention to use contraceptive methods around 46% of voucher users expressed their unwillingness to use contraceptives in the absence of a voucher scheme.

**Table S2: Contraceptive method mix for voucher users receiving family planning services through the GS multipurpose-purpose voucher scheme – 24 months period**

| **Method** | **Frequency (number of voucher clients)** | **Percentage** |
| --- | --- | --- |
| Pill | 33 | 8.1 |
| IUD | 126 | 30.8 |
| Injections | 92 | 22.5 |
| Implants | 1 | 0.2 |
| Condom | 80 | 19.6 |
| Periodic Abstinence | 2 | 0.5 |
| Withdrawal | 10 | 2.4 |
| Female sterilization | 11 | 2.7 |
| Male sterilization | 1 | 0.2 |
| Non user | 53 | 13.0 |
| Total | 409 | 100.0 |
